# ^13^C Incorporation as a Tool to Estimate Biomass Yields in Thermophilic and Mesophilic Nitrifying Communities

**DOI:** 10.3389/fmicb.2019.00192

**Published:** 2019-02-13

**Authors:** Tom G. L. Vandekerckhove, Samuel Bodé, Chaïm De Mulder, Siegfried E. Vlaeminck, Nico Boon

**Affiliations:** ^1^Center for Microbial Ecology and Technology, Ghent University, Ghent, Belgium; ^2^Isotope Bioscience Laboratory (ISOFYS), Ghent University, Ghent, Belgium; ^3^BIOMATH, Department of Mathematical Modelling, Statistics and Bioinformatics, Ghent University, Ghent, Belgium; ^4^Research Group of Sustainable Energy, Air and Water Technology, University of Antwerp, Antwerp, Belgium

**Keywords:** biological nitrogen removal, sensitivity analysis, *Nitrososphaera gargensis*, *Nitrospira*, observed yield

## Abstract

Current methods determining biomass yield require sophisticated sensors for *in situ* measurements or multiple steady-state reactor runs. Determining the yield of specific groups of organisms in mixed cultures in a fast and easy manner remains challenging. This study describes a fast method to estimate the maximum biomass yield (Y_max_), based on ^13^C incorporation during activity measurements. It was applied to mixed cultures containing ammonia oxidizing bacteria (AOB) or archaea (AOA) and nitrite oxidizing bacteria (NOB), grown under mesophilic (15–28°C) and thermophilic (50°C) conditions. Using this method, no distinction could be made between AOB and AOA co-existing in a community. A slight overestimation of the nitrifier biomass due to ^13^C redirection *via* SMP to heterotrophs could occur, meaning that this method determines the carbon fixation activity of the autotrophic microorganisms rather than the actual nitrifier biomass yield. Thermophilic AOA yields exceeded mesophilic AOB yields (0.22 vs. 0.06–0.11 g VSS g^-1^ N), possibly linked to a more efficient pathway for CO_2_ incorporation. NOB thermophilically produced less biomass (0.025–0.028 vs. 0.048–0.051 g VSS g^-1^ N), conceivably attributed to higher maintenance requirement, rendering less energy available for biomass synthesis. Interestingly, thermophilic nitrification yield was higher than its mesophilic counterpart, due to the dominance of AOA over AOB at higher temperatures. An instant temperature increase impacted the mesophilic AOB yield, corroborating the effect of maintenance requirement on production capacity. Model simulations of two realistic nitrification/denitrification plants were robust toward changing nitrifier yield in predicting effluent ammonium concentrations, whereas sludge composition was impacted. Summarized, a fast, precise and easily executable method was developed determining Y_max_ of ammonia and nitrite oxidizers in mixed communities.

## Introduction

Ammonium is a major reactive nitrogen species, accumulating in the environment due to anthropogenic distortion of the nitrogen cycle (Steffen et al., 2015). In Flanders for example, 28% of the released nitrogen ends up in surface water (Coppens et al., 2016). This accumulation can cause eutrophication, resulting in hypoxia and eventually fish mortality (Camargo and Alonso, 2006). To mitigate this reactive nitrogen pollution, biological wastewater treatment is widely used to treat wastewater before discharge. Nitritation and nitrification (nitritation+nitratation) play a key role in the removal of ammonium in wastewater and entails the microbial oxidation of ammonia to nitrite and nitrate respectively (Metcalf and Eddy, 2003; Vlaeminck et al., 2012). Ammonia oxidizing bacteria (AOB) and archaea (AOA) catalyze the conversion of ammonia (NH_3_) to nitrite (NO_2_^-^), called nitritation, and is typically the rate-limiting step. Subsequently, nitrite oxidizing bacteria (NOB) further oxidize nitrite to nitrate, also known as nitratation.

The biomass yield (Y) plays a vital role in the design, operation and modeling of a biological wastewater treatment facility and represents the amount of biomass produced relative to the amount of substrate removed. A distinction should be made between observed yield (Y_obs_) and maximum yield (Y_max_). The observed yield is the net effect of both growth and decay/maintenance of biomass, whereas the maximum yield is higher, and only includes growth, as obtained immediately upon oxidation of ammonium or nitrite, in the case of nitrifiers (Metcalf and Eddy, 2003). The Y_obs_ can be determined *in situ*, for example, by using cumulative terms over several days but makes no distinction between AOB/AOA and NOB in a mixed culture (Courtens et al., 2016b). This is interesting for the operation of a treatment plant, for it dictates the actual sludge production and thus its disposal cost, a major cost factor in water treatment. The Y_max_ on the other hand is key for the design and modeling of wastewater treatment. An estimation of the maximum biomass yield could be made based on stoichiometry or thermodynamic principles of biological reactions. This, however, requires an assumption for the biomass stoichiometric formula (Metcalf and Eddy, 2003). When short-term tests are performed for the determination of biomass yield, it is often assumed that biomass decay can be neglected and the maximum yield is obtained. Respirometry could be used to calculate the Y_max_ from the area under the respirogram and the substrate concentration added (Beccari et al., 1979; Vogelaar et al., 2003). Respirometric measurements could also be integrated with titrimetric measurements through the use of a Titrimetric and Off-Gas Analyzer (TOGA) (Blackburne et al., 2007a). Furthermore, a model could be fitted to transient data obtained in several steady-state reactors, where the kinetic parameters (including Y_max_) were acquired by non-linear regression analysis (Gee et al., 1990). The proposed methods are valid, but require either the use of sophisticated sensors for *in situ* measurements or the laborious operation of multiple reactors in steady state. Determining the yield of specific groups of organisms in mixed cultures in a fast and easy manner remains challenging.

In literature, AOB yields range from 0.06 to 0.3 g volatile suspended solids (VSS) g^-1^ N, whereas an AOA yield of 0.09 g dry weigh g^-1^ N has been determined only once (Table 3). Since it involved a pure culture of AOA, the dry mass (or total suspended solids, TSS) can be assumed to approximate the volatile suspended solids. The biomass yield of NOB ranges from 0.042–0.15 g VSS g^-1^ N solids (Table 4). Nitrifiers are autotrophic organisms, requiring energy for cell synthesis from CO_2_ (Konneke et al., 2014). Heterotrophs, on the other hand, derive the necessary building blocks from their metabolic pathway (Madigan, 2009). Autotrophs, thus spend a great deal of the available energy for synthesis, rendering a substantially lower biomass yield compared to heterotrophic bacteria (0.47 g VSS g^-1^ COD; Henze et al., 2000).

In modeling nitrification/denitrification systems, the Y_max_ value is typically assumed constant and lumped for AOB/AOA and NOB. In the activated sludge models (ASM), for example, the value (Y_A_) is set at 0.17 g VSS g^-1^ N (Henze et al., 2000). However, the energy derived from nitrogen oxidation is used in both maintenance and biomass assimilation. Energy requirement for maintenance might be influenced by changing environmental conditions, causing fluctuations in the yield. Indeed, changing pH conditions rendered slightly different values for Y_max_ (Blackburne et al., 2007a). It is thus important to assess the influence of these changes on the modeling of a wastewater treatment plant.

In this study, a fast and easy method estimating the maximum yield of nitrifying organisms (AOB, AOA, and NOB) was developed and applied to a mesophilic nitrifying mixed community at two different temperatures and to two thermophilic nitrifying mixed communities for, to the authors knowledge, the first determination of nitrifying yield in mixed cultures at elevated temperatures (50°C). The method was based on the incorporation of ^13^C, provided as H^13^CO_3_^-^, during the oxidation of ammonium or nitrite in a short-term activity test. Isotopically labeled carbon has been used to determine the autotrophic nature of nitrifying organisms by the incorporation into biomarker molecules, but never for Y_max_ determination (Kim et al., 2012; Courtens et al., 2016a). To evaluate the impact of changing Y_max_ of nitrification on the modeling of a nitrification/denitrification installation, a municipal and an industrial wastewater treatment plant case study was simulated at changing autotrophic yields.

## Materials and Methods

### Test Set-Up

To determine the maximum yield of AOB, AOA, and NOB, ex-situ activity measurements were performed using NaH^13^CO_3_ as carbon source. By monitoring the incorporation of ^13^C into the total biomass and the oxidation of ammonium or nitrite, Y_max_ could be derived.

Serum flasks of 120 mL were utilized, containing 50 mL mixed liquor and buffer solution at pH 7.3 with a final concentration of 50 mg NH_4_^+^- or NO_2_^-^-N L^-1^, 0.3 g P L^-1^ (KH_2_PO_4_/K_2_HPO_4_), 0.5 g (99%) NaH^13^CO_3_ L^-1^, 0.2 g MgSO_4_x7H_2_O L^-1^, 0.1 g CaCl_2_ L^-1^ and 0.1 mL L^-1^ of trace elements (Kuai and Verstraete, 1998). Prior to the addition of biomass to the serum flask, the sludge was washed several times using buffer medium devoid of bicarbonate and substrate to wash away any remaining unlabeled HCO_3_^-^ or substrate. During the washing procedure, sedimentation at the imposed test temperature rather than centrifugation was used to avoid environmental or physical shocks. Biomass concentration was determined using the concentration in the washed and homogenized inoculum sample (executed in triplicate) and the imposed dilution in the serum flasks. In order to limit the volume needed for ^13^C analysis (±1.5 mL), the used biomass concentration was set around 1 g VSS L^-1^. For each inoculum, the carbon content was measured experimentally.

Different inocula were used, one mesophilic and two thermophilic nitrifying communities (Table 1). One thermophilic inoculum was taken from a “constant temperature MBR,” originating from compost samples and enriched in a bioreactor (Courtens et al., 2016a). Another thermophilic inoculum, called “temperature increase MBR,” emanated from a mesophilic nitrifying bioreactors upon which a temperature increase was imposed until thermophilic conditions were reached (Courtens et al., 2016b). Both thermophilic communities contained *Nitrososphaera gargensis*-like AOA, no AOB and *Nitrospira calida*-like NOB (Courtens et al., 2016a,b). For the mesophilic inoculum, taken from a municipal wastewater treatment plant (Ghent, Belgium), two temperatures were tested in parallel to evaluate the effect on the maximum AOB and NOB yield (15 and 28°C).

**Table 1:** Different nitrifying inocula used for the determination of AOB/AOA and NOB yield, the temperature of their origin and the temperature at which the yield was determined.

Origin	Site temperature (°C)	Test temperature (°C)	Reference
Municipal wastewater treatment plant Ossemeersen (aerobic basin)	±15^∗^	15	/
Municipal wastewater treatment plant Ossemeersen (aerobic basin)	±15^∗^	28	/
Constant temperature MBR^∗∗^	50	50	Courtens et al., 2016a
Temperature increase MBR^∗∗^	50	50	Courtens et al., 2016b

*^∗^Sample taken in April/May in Ghent, Belgium. ^∗∗^After publication, these reactors were transformed to MBR reactors for the evaluation of kinetic parameters.*

The inocula were incubated with ammonium and nitrite as substrate in parallel to enable the differentiation between the AOB/AOA and NOB yield. All tests were performed in triplicate on a temperature controlled shaker (120 rpm). The serum flasks were closed with rubber stoppers to prevent excessive intrusion of natural CO_2_ from the atmosphere. During the incubation, three to five liquid samples were taken over time from the homogenized mixed liquor for ammonium, nitrite and ^13^C analysis. Samples for ammonium and nitrite measurement (0.5 mL) were filtered over a 0.2 μm filter. Biomass samples (1.5 mL) for ^13^C analysis were centrifuged at 14,000 rpm for 5 min, supernatants was removed and the pellet was solubilized in a buffer medium at pH 5 with a final concentration of 0.2 g P L^-1^ (KH_2_PO_4_/K_2_HPO_4_). This procedure was repeated 3 times to remove the remaining solubilized H^13^CO_3_^-^ in the biomass sample. After removing the supernatants for the last time, the pellet was stored in a freezer at -20°C, freeze-dried and analyzed for the abundance of ^13^C (see section Chemical Analyses).

In order to assess whether other autotrophic pathways contributed to ^13^C incorporation under the imposed conditions, a control experiment was conducted. The mesophilic and thermophilic nitrifying community was tested at 28 and 50°C respectively. Similar incubations (in triplicate) were performed as described above with the same sampling procedure, but ammonium and nitrite were provided in the same incubation (45–80 mg N L^-1^). Both ammonium oxidation and nitrite oxidation was inhibited by the addition of 0.5 g ATU L^-1^, 1.5 g ATU L^-1^ and 10 mM chlorate for AOB, AOA, and NOB respectively. Under these conditions, no nitrification occurred, rendering other autotrophic pathways responsible for ^13^C incorporation.

### Validation of Optimal Experimental Conditions

To ensure that oxygen was not limiting, a validation experiment with the thermophilic inocula was executed. A serum flask activity test as described above (with natural NaHCO_3_ and without sampling for ^13^C analysis) was compared to a parallel activity measurement in a 96 well plate as described and validated before (Courtens et al., 2016a,b). One well represented one incubation, with a working volume of 250 μL, in which medium and biomass are mixed. The activity measurements in serum flasks and 96 Well plate were executed at the same time. Buffer medium for the 96 well activity measurements and serum flask activity test was the same. The 96 Well plate was shaken at 50°C and 600 rpm in a Thermoshaker (Hangzhou Allsheng Instruments, Hangzhou, China). Liquid samples (2 μL) were taken from each well incubation for ammonium and nitrite analysis to determine the ammonium and nitrite oxidation rate. Serum flask and 96 Well plate activity measurements were performed in triplicate and sextuple respectively.

### Calculations to Determine the Yield Factor

In order to acquire a yield value in conventional engineering units of g VSS g^-1^ N, some calculations were required. After isotope analysis, the abundance of ^13^C in the biomass sample was known.

#### Biomass Production

Knowing the abundance of ^13^C (%) at every time point, the fraction of new biomass at time point n (f_n_) can be calculated (1).

(1) $$f_{n}=\frac{a^{13}C_{tn}\;-\;a^{13}C_{tn-1}}{a^{13}C_{medium}\;-\;a^{13}C_{tn-1}}$$

With a^13^C_tn_ the fraction of ^13^C at time point n, a^13^C_tn-1_ the fraction of ^13^C at time point n-1 and a^13^C_medium_ the purity of ^13^C in the medium (in this case 99%) to account for growth on the limited amount of ^12^C available.

Using the fraction of new biomass at every time point (f_n_), the growth factor (f_g_) was calculated (2).

(2) $$f_{g}=\frac{f_{n}}{1\;-\;f_{n}}$$

After determining the growth factor, the biomass concentration at every time point was acquired (3).

(3) $${\left[VSS\right]}_{tn}={\left[VSS\right]}_{tn-1}+f_{g}*{\left[VSS\right]}_{tn-1}$$

With [VSS]_tn_ the biomass concentration at time point n, [VSS]_tn-1_ the amount of biomass at time point n-1.

#### Biomass Yield

The biomass concentration could be plotted as a function of the nitrogen concentration, to which a straight line was fitted, with the Y_max_ as slope. The average and standard deviation of the three replicates resulted in the variation. Performing this experiment and these calculations using ammonium as substrate resulted in the combined yield of AOB/AOA and NOB. As incubations of an inoculum with ammonium and nitrite were performed in parallel, subtracting the yield obtained by using nitrite as substrate (NOB yield) from the yield obtained by using ammonium as substrate yielded the separate yield for AOB/AOA.

### Molecular Analyses

#### DNA Extraction and Quality Control

To confirm that AOB were highly dominant over AOA in the mesophilic inoculum, samples were taken for 16S rRNA gene amplicon sequencing and qPCR. Samples were stored at -20°C prior to DNA extraction. DNA was extracted using the ZymoBIOMICS DNA Microprep Kit (Zymo Research, United States) according instructions of the manufacturer. Quality assessment of the DNA extracts was performed by visualization with ethidium bromide in a 2% agarose gel (120 V, 20 min) after which the concentration was measured fluorometrically using the QuantiFluor^®^ dsDNA System (Promega, United States). For the DNA extracts for 16S rRNA gene amplicon sequencing, Illumina 16S rRNA gene amplicon libraries were generated and sequenced by BaseClear BV (Leiden, The Netherlands). The DNA extracts were sent to Baseclear B.V. for 16S rRNA gene amplicon sequencing on the Miseq platform for both bacteria and archaea. The sequencing data are deposited at the NCBI (National Center for Biotechnology Information) database under accession number SRP173880.

#### 16S rRNA Gene Amplicon Sequencing on the Miseq Platform

Barcoded amplicons from the V3 to V4 region of the16S rRNA genes were produced using a 2-step PCR. Subsequently, 10–25 ng genomic (g)DNA was used as template for the first PCR (25 cycles at 55°C), with a total volume of 50 μL using the 341F (5′-CCTACGGGNGGCWGCAG-3′) and the 785R (5′-GACTACHVGGGTATCTAATCC-3′) primers for bacteria and the 518F (5′-CAGCMGCCGCGGTAA-3′) and 905R (5′-CCCGCCAATTCCTTTAAGTTTC-3′) primers for archaea, supplemented with Illumina adaptor sequences. The PCR products were purified using Ampure XP beads according to the manufacturer’s instructions, after which the size was checked on a Fragment analyzer (Advanced Analytical). Quantification was done by means of fluorometric analysis. For the second PCR (6 cycles at 55°C), purified PCR products were combined with sample-specific barcoded primers (Nextera XT index kit, Illumina). Subsequently, After purifying the PCR products, they were checked on a Fragment analyzer (Advanced Analytical) and quantified. PCR amplicons were generated using in-house protocols at BaseClear and purified using Ampure XP beads according to the manufacturer’s instructions. Subsequently, multiplexing, clustering, and sequencing was performed on an Illumina MiSeq with the paired-end (2x) 300 bp protocol and indexing. The sequencing run was analyzed using the Illumina CASAVA pipeline (v1.8.3) with demultiplexing based on sample-specific barcodes. The resulting raw sequencing data was processed by removing the sequence reads of low quality (only “passing filter” reads were selected). Reads containing adaptor sequences or PhiX control were discarded with an in-house filtering protocol. Quality assessment of the remaining reads was performed by the FASTQC quality control tool version 0.10.0.

Read assembly and cleanup was largely executed using previously described guidelines (Schloss et al., 2011; Kozich et al., 2013). Mothur (v.1.40.3) was used to assemble reads into contigs, perform alignment-based quality filtering (alignment to the mothur-reconstructed SILVA SEED alignment, v. 128), remove chimeras, assign taxonomy using a naïve Bayesian classifier (Wang et al., 2007) and SILVA NR v132 and cluster contigs into OTUs at 97% sequence similarity. All sequences classified as Eukaryota, Archaea (or Bacteria when archaea were sequenced), Chloroplasts and Mitochondria were removed. Also, if sequences could not be classified [even at (super)Kingdom level] they were removed. For each OTU, representative sequences were selected as the most abundant sequence within that OTU.

#### Total Bacteria and Archaea as Determined by qPCR

After DNA extraction, extracts were diluted 10- or 20-fold, yielding a final DNA concentration between 1 and 10 ng μL^-1^. For each sample, real-time PCR (qPCR) for bacteria and archaea was executed in triplicate on a StepOnePlus^TM^ Real-Time PCR System (Applied Biosystems, Carlsbac, CA, United States). The reaction mixture contained 10 μL of GoTaq^®^ PCR Master Mix, 3.5 μL of nuclease-free water, and 0.75 μL of each primer (from a 10 μM stock concentration), and 5 μL of template DNA. To quantify total bacteria, the general bacterial primer P338F (5′-ACTCCTACGGGAGGCAGCAG) and P518R (5′-ATTACCGCGGCTGCTGG) was used (Ovreas et al., 1997). The qPCR program for the quantification of total bacteria consisted of a denaturation step (10 min at 94°C), followed by 40 cycles of denaturation (15 s at 94°C) and a combined annealing/extension (1 min at 60°C). To quantify total archaea, the general archaeal primers ARC787F (5′-ATTAGATACCCSBGTAGTCC) and ARC1059R (5′-GCCATGCACCWCCTCT) were used (Yu et al., 2005). A similar qPCR program was applied as described above for bacteria, except for a 10 s denaturation time instead of 15 s. The overall quality of the qPCR was validated based on different parameters obtained during analysis with the StepOnePlus software V2.3.

### Chemical Analyses

Total suspended solids (TSS) and volatile suspended solids (VSS) were measured according to standard methods (APHA, 1992). Liquid samples for ammonium and nitrite determination were analyzed spectrophotometrically with the Berthelot and Montgomery reaction, including a triplicate standard curve for each analysis run. Measurements were obtained using a Tecan infinite plate reader (Tecan, Switzerland). The bulk ^13^C abundance was determined with an elemental analyzer (ANCA SerCon, Crewe, United Kingdom) coupled a isotope ratio mass spectrometry (IRMS) detector (20-22 SerCon, Crewe, United Kingdom), with a high precision (errors in the range of 0.0005%).

### Statistical Analysis

Statistical analysis was applied to check for significant differences in activity between the serum flasks and the 96 Well plate, significant differences in the obtained yield and significant differences in yield during the sensitivity analysis. Prior to testing the null hypothesis, the data was screened and explored with boxplots. Normality was examined visually using normal QQ-plots and as a formal normality hypothesis test, a Shapiro Wilks test on the residuals was applied. The homogeneity of variances was checked with the Bartlett test. If normality and homoscedasticity could be assumed, the null hypothesis was tested with a one-way ANOVA. Pairwise differences or contrasts were tested with Tukey. In case that normality could not be assumed, the null hypothesis was tested with a Kruskal Wallis rank sum test (non-parametric test) instead of one-way ANOVA. Pairwise Wilcoxon Rank Sum Tests with Holm correction for multiple testing were applied to determine the pairwise difference. All formal hypothesis tests were conducted on the 5% significance level (α = 0.05), except for the homogeneity of variances (1%). All statistical analysis were executed in R version 3.3.1 (2016-06-21) on an x86_64-w64-mingw32/x64 (64-bit) platform running under Windows 8.1 Enterprisex64 (build 9600).

### Implication for Modeling

To assess the impact of different autotrophic yield values on biomass composition and effluent concentrations, a couple of simple scenarios were simulated with two different models representing two distinct nitrification/denitrification case studies. The autotropic yield values (Y_A_) used in these simulations varied between 0.05 and 0.5 g cell COD g^-1^ N, with steps of 0.05 (default yield is 0.24 g cell COD g^-1^ N). This resulted in a total of 10 scenarios to compare per case study.

The first case was based on information received from an installation treating potato wastewater and was simulated using a simplified model layout (Table 2). The activated sludge model was ASM1 (Henze et al., 2000), the model for the Secondary Settling Tank (SST) was the Takacs model (Takacs et al., 1991). Any changes made to the default model values aim to represent a realistic situation and stem from a lack of sufficient information on the real installation. For example, as no details were available on the membrane separation that is in place in reality, the Sludge Volume Index (SVI) was adjusted to 1 to mimic the real situation.

**Table 2:** Model specifics of the two considered cases.

Parameter	Potato wastewater	Municipal wastewater	Unit
Tank volume	13,889	51,757	m^3^
Influent flow rate	2160	165,343	m^3^ d^-1^
Return Activated Sludge	6823	*Controlled*	m^3^ d^-1^
Waste Sludge flow rate	500	*Controlled*	m^3^ d^-1^
SVI	1	*NA*	mL g^-1^
Influent COD concentration	2109	400	g m^-3^
Influent Total Nitrogen concentration	355	39	g N m^-3^

*Controlled indicates that the variable is controlled and no one value can be assigned. NA indicates that the variable is not applicable to the case.*

The second case was the Eindhoven Wastewater Treatment Plant (WWTP), operated by Waterboard De Dommel (The Netherlands). This 750,000 IE WWTP has been the subject of years of research and is thus modeled in large detail (Cierkens et al., 2012; Amerlinck et al., 2016). In the current context, the latest calibrated version of the model was used.

All scenarios were simulated as a steady state operation (i.e., constant influent flow and concentrations) for a period of 150 days in the WEST simulation environment (MIKE Powered by DHI, Denmark). In the case of the potato wastewater, the values of the state variables used for comparison are the values obtained at the end of a steady state simulation, thus representing the steady state of the simulated installation. For the municipal wastewater, the average of the last 10 days of steady state simulation was taken. This was necessary because control algorithms in the model caused fluctuations in simulation results (also in steady state) and the last simulated value therefore does not necessarily represent an actual steady state.

## Results and Discussion

### Thermophilic AOA Produce More Biomass Than Mesophilic AOB Whereas Thermophilic NOB Produce Less Biomass Than Mesophilic NOB

**FIGURE 1:**
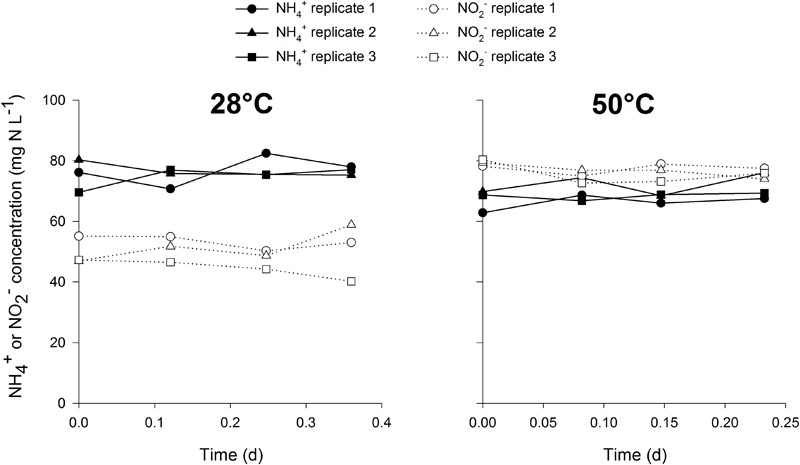
Ammonium and nitrite concentrations in the closed serum flasks (*n* = 3) at 28°C (left) and 50°C (right) during the control activity measurements in which nitrification was inhibited.

**FIGURE 2:**
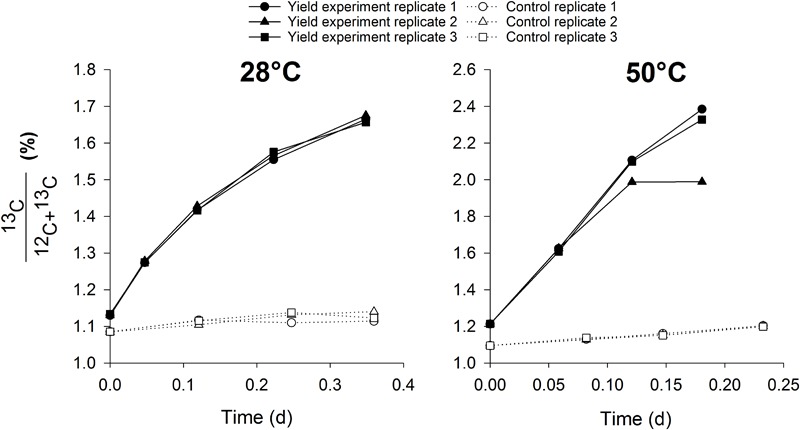
The increase of ^13^C abundance in the biomass during the control activity measurements in which nitrification was inhibited (dotted lines, *n* = 3) and during the actual yield experiments with ammonium in which nitrification was not inhibited (full lines, *n* = 3).

This methodology has a distinct advantage of mainly targeting a specific group in mixed cultures, rendering no need for enrichment or isolation of the target organisms. The mixed cultures tested in this study contained different abundances of nitrifiers, with the thermophilic inocula much more enriched than the mesophilic cultures. This shows the wide applicability of this method to estimate nitrifier biomass yield in mixed cultures with low or high abundance of nitrifiers. The methodology might even be extendable to other groups than AOB, AOA or NOB, for example methane oxidizing or anammox bacteria. By using specific growth conditions for one target microbial group whilst providing the necessary carbon source with a stable isotope, growth can be estimated in small scale, easily executable and fast activity measurements. In this case, the biomass was washed prior to the test to remove the unlabeled bicarbonate present in the mixed liquor, along with any other organic carbon source. The experiments were conducted aerobically with the addition of bicarbonate and ammonium or nitrite. Under these conditions, mostly nitrifiers would be active and growing, rendering other autotrophic pathways for ^13^C incorporation negligible. To confirm this hypothesis, control incubations at 28 and 50°C were performed with the mesophilic and thermophilic inoculum respectively, in which nitrification was inhibited in the presence of ammonium, nitrite and ^13^C. Ammonium and nitrite oxidation did not occur in the control experiment (Figure 1), but there was a slight increase in ^13^C abundance in the biomass, possibly attributed to other autotrophic pathways than nitrification. However, the increase in the control experiment was relatively low, 6–8% of the increase observed when nitrification was not inhibited in the presence of ammonium (Figure 2). Also, the contribution of ^13^C incorporation not attributed to nitrification was similar in both inocula, meaning that the comparison of the obtained mesophilic and thermophilic yields is not affected, only the absolute values are affected (6–8% overestimation). To more accurately determine the nitrifier biomass yield, this method could be performed on a pure culture of AOA, AOB and NOB at different cultivation temperatures.

Heterotrophic incorporation of ^13^C would only be possible from feeding on soluble microbial products (SMP), leached during nitrifier decay (biomass-associated products) or substrate metabolism (utilization-associated products) (Rittmann et al., 1994; Barker and Stuckey, 1999). Due to the short duration of the experiments (4–21 h) and the low decay rate of nitrifiers (0.03–0.06 d^-1^; Henze, 1997), the decay rate was neglected, as is often done in short-term experiments. Nonetheless, ^13^C incorporated by heterotrophic bacteria derived from nitrifier decay products was originally incorporated by nitrifiers and, thus, was part of the nitrifier biomass. Furthermore, decay products from nitrifiers would contain more ^12^C than ^13^C, limiting the impact on overall ^13^C incorporation. Using the range of decay rates and the ^13^C content of the biomass during the incubations, the contribution of heterotrophic growth on decay products of nitrifiers to the total biomass production was theoretically estimated to be 0.17–0.39%. Oxidation of utilization-associated products, released during the metabolism of nitrifiers, and the concomitant growth of heterotrophic bacteria occurs from 4 to 6 h of incubation without organic carbon (Kindaichi et al., 2004; Okabe et al., 2005). Considering the duration of each incubation (4 h at 50°C, 8 h at 28°C and 21 h at 15°C), ^13^C redirection *via* SMP to heterotrophs probably did not occur at 50°C but could not be completely excluded from the mesophilic experiments. However, the linear trend of the ^13^C incorporation (Figure 4) suggested that no noticeable additional incorporation occurred from 4 to 6 h of incubation. It is, thus, most likely that nitrifiers were responsible for the observed ^13^C incorporation. Nonetheless, a slight overestimation of the nitrifier biomass due to this heterotrophic contribution could occur, meaning that this method determines the carbon fixation activity of the autotrophic microorganisms rather than the actual nitrifier biomass yield. This method should, thus, be seen as a swift estimation of the nitrifier biomass yield rather than an exact determination of its value, as a slight overestimation is possible.

**FIGURE 3:**
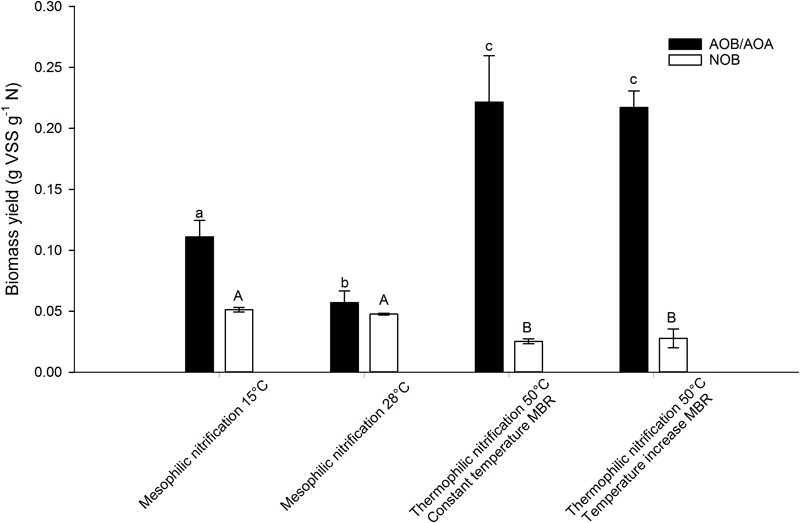
AOB/AOA and NOB yield of a mesophilic nitrifying inoculum at 15 and 28°C and of two thermophilic nitrifying communities at 50°C, determined based on the incorporation of ^13^C. Error bars represent the standard deviation of triplicate incubations with the same inoculum (technical replicates). Significant pairwise differences (*p* < 0.05) of AOB/AOA yield are indicated with different non-capital letters, significant pairwise differences (*p* < 0.05) of NOB yield are indicated with different capital letters.

**FIGURE 4:**
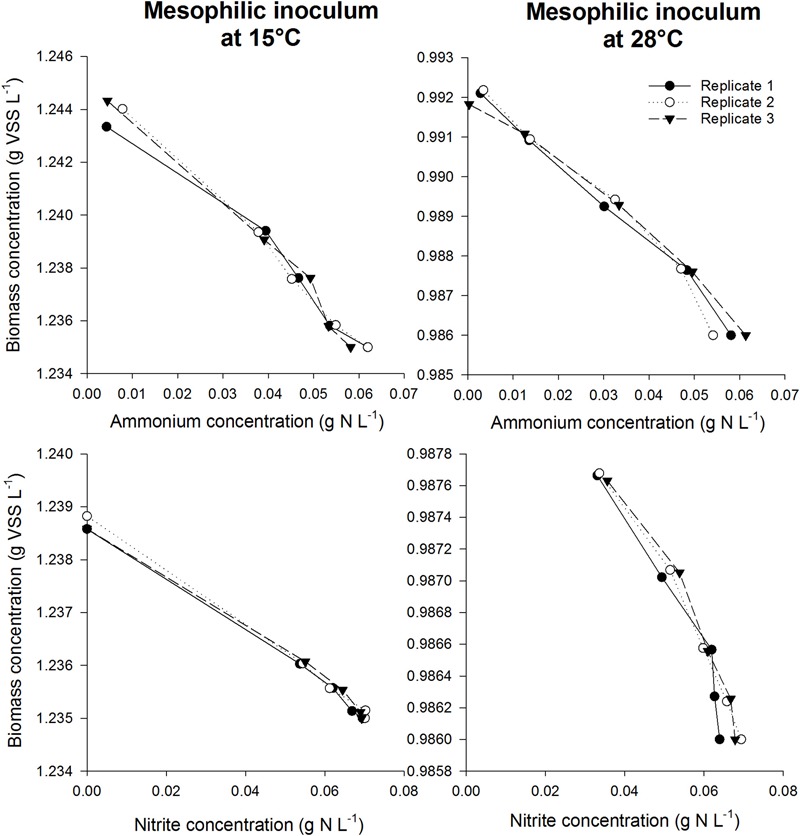
Biomass concentration as a function of ammonium concentration (top) or nitrite concentration (bottom) during the incubations for the mesophilic inoculum at 15°C (left) and 28°C (right). The slope was used to determine the biomass yield for NOB in case nitrite was added as substrate, whereas the total AOB and NOB yield was obtained using the slope in case ammonium was added as substrate. By subtracting the NOB yield from the total yield, the AOB yield could be calculated.

**FIGURE 5:**
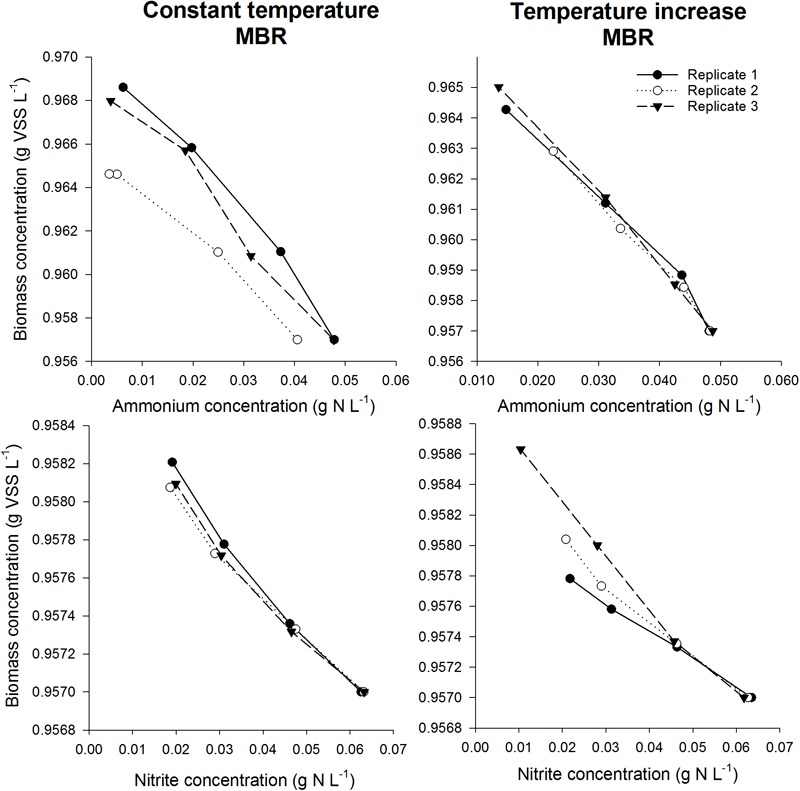
Biomass concentration as a function of ammonium concentration (top) or nitrite concentration (bottom) during the incubations for the thermophilic inoculum in the constant temperature MBR (left) and the temperature increase MBR (right). The slope was used to determine the biomass yield for NOB in case nitrite was added as substrate, whereas the total AOA and NOB yield was obtained using the slope in case ammonium was added as substrate. By subtracting the NOB yield from the total yield, the AOA yield could be calculated.

After validating that the experimental conditions were optimal and non-limiting conditions prevailed in the serum flask activity measurement, the biomass yield of different nitrifying inocula was determined based on the incorporation of ^13^C (Figure 3). In order to assess the impact of temperature on the yield parameter, two different temperature conditions were imposed on the mesophilic nitrifying biomass (15 and 28°C). In all experiments, a linear relationship (*R*^2^> 92%) was observed between the biomass produced and the nitrogen oxidized (Figure 4, 5).

The tested thermophilic communities harbored AOA (related to *Nitrososphaera* gargensis) and were devoid of AOB due to the temperature stress (Courtens et al., 2016a,b). To our knowledge, the biomass yield of AOA has hardly been determined. Only one study, based on cell counts and protein measurements during the growth of *Nitrosopumilus maritimus* strain SCM1 at 28°C, showed a yield of 0.09 g dry mass g^-1^ N (Konneke et al., 2014). Since it involved a pure culture, the dry mass (or total suspended solids, TSS) can safely be assumed to approximate the volatile suspended solids. By doing so, the yield is much lower than the yields observed in this study (0.22 ± 0.04 and 0.22 ± 0.01 g VSS g^-1^ N for the constant temperature and temperature increase MBR respectively). This difference might be due the thermophilic nature of the AOA in this study. As little is known about AOA biomass yields, further research on AOA-containing mixed communities, cultivated at different temperatures, can provide more insight.

When comparing the thermophilic AOA yields to mesophilic AOB yields described in literature, they fall within the wide range of reported values (0.06–0.3 g VSS g^-1^ N) (Table 3). In this study, however, the same method was applied on a mesophilic AOB containing mixed culture and a thermophilic AOA containing mixed culture, revealing that the thermophilic AOA yield exceeded the mesophilic AOB yield (0.11 ± 0.01 and 0.06 ± 0.01 g VSS g^-1^ N at 15 and 28°C respectively). To confirm that AOB were highly dominant over AOA in the mesophilic inoculum, samples were taken for 16S rRNA gene amplicon sequencing and qPCR. The qPCR results and the biomass concentration of the samples from the mesophilic biomass were combined to yield total bacteria and archaea in copies g^-1^ VSS. Together, they quantitatively represented the total community. When considering the relative abundance of ammonium oxidizing bacteria (AOB) and nitrite oxidizing bacteria (NOB) in the bacterial community and ammonium oxidizing archaea (AOA) in the archaeal community, as determined by 16S rRNA gene amplicon sequencing, an estimation was made of the relative abundance of each microbial group in the mesophilic biomass. The results should be interpreted with care. Although the specificity is high, it is not perfect due to an unequal coverage by the primer sets. The tandem qPCR and 16S rRNA gene amplicon sequencing offers semi-reliable estimates of quantified abundance, but other techniques (such as FISH or qPCR with specific primers) might provide more reliable quantification. In the bacterial community, 0.66% of the reads were affiliated with AOB and 2.55% with NOB. In the archaeal community, 0.45% of all reads were affiliated with AOA. The qPCR analysis revealed that 99.97 ± 11.45% of all 16S gene copy number (archaeal + bacterial) were bacterial of nature (3.5∗10^13^ ± 1.1∗10^13^ copies g^-1^ VSS for Bacteria and 8.7∗10^9^ ± 1.6^∗^10^9^ copies g^-1^ VSS for Archaea). Combining these results yielded a relative abundance of AOA that was negligible compared to AOB (0.0001 ± 0.00001% vs. 0.67 ± 0.13% respectively). When it comes to energy efficiency of the CO_2_ fixation pathway, AOA score better than AOB as the thaumarchaeal HP/HB cycle requires about a third less energy than the Calvin-Benson cycle utilized by AOB (Dworkin et al., 2006). Also, CO_2_ fixation by AOA is not accompanied by losses caused by the oxygenase side-reaction of ribulose-1,5-bisphosphate carboxylase/oxygenase, leading to an additional loss of about 20% of fixed carbon in the Calvin-Benson cycle. Lastly, AOA ribosomal content and overall cell volume is smaller compared to AOB. These factors could explain the higher yield of AOA compared to AOB, as more energy can be redirected to biomass production. In a previous study, comparing the biomass yield of *N. maritimus* and *N. oceani*, a 1.5 times higher AOA yield was observed (Konneke et al., 2014).

**Table 3:** Summary of reported maximum yield values of AOB and AOA with the species, imposed temperature and method applied.

	Species	Temperature (°C)	Yield (g VSS g^-1^ N)	Method	Reference
AOB	/	21	0.13	Model fit to respirometric data	Katipoglu-Yazan et al., 2015
	*Nitrosomonas*	23	0.3	Model fit to respirometric data	Gee et al., 1990
	*Nitrosomonas europaea*	30	0.18	non-linear regression of steady state data from chemostat	Kantartzi et al., 2005
	/	/	0.14–0.20	Model fit to respirometric data	Pai et al., 2010
	*Nitrosomonas*	21	0.14	Titrimetric and off-gas analysis	Blackburne et al., 2007a
	*Nitrosomonas*	20	0.15	Model fit to respirometric data	Beccari et al., 1979
	*Nitrosococcus oceani*	28	0.06^∗^	Growth in unbuffered synthetic medium	Konneke et al., 2014
	/	20	0.10–0.12	Literature summary	Henze, 1997
	/	15	0.11	^13^C incorporation	This study
	/	28	0.06	^13^C incorporation	This study
AOA	*Nitrosopumilus maritimus*	28	0.09^∗^	Growth in unbuffered synthetic medium	Konneke et al., 2014
	*Nitrososphaera gargensis*	50	0.19–0.22	^13^C incorporation	This study

*/, not determined. ^∗^g dry weight g^-*1*^ N.*

As representatives of NOB, the tested thermophilic reactors were populated by *Nitrospira calida* related organisms (Courtens et al., 2016a,b). All thermophilic NOB described up to date are related to *Nitrospira calida*, with one study reporting the presence of *Nitrospira moscoviensis* in co-culture with *Nitrospira calida* (Lebedeva et al., 2011; Marks et al., 2012; Edwards et al., 2013). However, the biomass yield of thermophilic NOB has not been determined yet. In this study, relatively low biomass yields were found compared to literature of mesophilic NOB, namely 0.025 ± 0.002 and 0.028 ± 0.007 g VSS g^-1^ N for the constant temperature and temperature increase MBR respectively (Table 4). They were also lower than the NOB yield obtained for the mesophilic biomass (0.051 ± 0.002 and 0.048 ± 0.001 g VSS g^-1^ N at 15 and 28°C respectively), which were in accordance with literature.

**Table 4:** Summary of reported maximum yield values of NOB with the species, imposed temperature and method applied.

Species	Temperature (°C)	Yield (g VSS g^-1^ N)	Method	Reference
*/*	21	0.042	Model fit to respirometric data	Katipoglu-Yazan et al., 2015
*Nitrobacter*	23	0.083	Model fit to respirometric data	Gee et al., 1990
*/*	22	0.042	Model fit to respirometric data	Ni et al., 2008
*Nitrobacter*	21	0.072	Titrimetric and off-gas analysis	Blackburne et al., 2007a
*Nitrospira*	22	0.15	Titrimetric and off-gas analysis	Blackburne et al., 2007b
*Nitrobacter*	22	0.049	Model fit to respirometric data	Vadivelu et al., 2006
*Nitrobacter winogradski*	30	0.058	non-linear regression of steady state data from chemostat	Kantartzi et al., 2005
*Nitrospira*	22	0.099	Model fit to respirometric data	Park et al., 2017
*/*	25.2	0.056	Model fit to respirometric data	Jubany et al., 2005
*/*	20	0.06	Literature summary	Henze, 1997
*/*	15	0.051	^13^C incorporation	This study
*/*	28	0.048	^13^C incorporation	This study
*Nitrospira calida*	50	0.025	^13^C incorporation	This study

*/, not determined.*

Interestingly, the overall nitrification yield was higher at thermophilic temperatures (0.24–0.25 g VSS g^-1^ N) compared to the mesophilic temperatures (0.16 and 0.10 g VSS g^-1^ N at 15 and 28°C respectively). This is a consequence of the AOA dominance over AOB at higher temperatures, as no AOB were found in the thermophilic inocula (Courtens et al., 2016a,b).

### Temperature Affects the AOB and NOB Yields

Temperature had an impact on the biomass yield of the mesophilic inoculum. At 28°C, the AOB yield of the activated sludge was only about half of the yield acquired at 15°C (Figure 3). No significant difference (*p* > 0.05) in NOB yield between 15 and 28°C was observed, whereas the NOB yield at 50°C was about half of the yield at mesophilic temperatures. Deriving a temperature effect from the latter observation is tricky, as it concerns different types of biomass with different NOB species. The NOB in the thermophilic biomass were related to *Nitrospira* (Courtens et al., 2016a,b), whereas the mesophilic biomass contained both *Nitrospira*- and *Nitrotoga*-related NOB (resp. 2.5 ± 0.1% and 1.0 ± 0.1% relative abundance in the total community). In general, the biomass yield of *Nitrospira* cultures is reported to be higher than *Nitrobacter* and *Nitrotoga* species (Nowka et al., 2015). Nonetheless, a lower yield at 50°C was obtained compared to 15 and 28°C.

These findings might indicate that temperature plays an important role in the maximum biomass production of nitrifying organisms. As the temperature in the wastewater treatment plant at the time of the sampling was about 15°C, an incubation at 28°C imposed a temperature shock to the organisms. This shock might cause the need for more energy investment in maintenance rather than biomass production for the AOB. Similarly, the NOB at 50°C might invest more energy in maintenance compared to the NOB in the mesophilic inoculum at 15 and 28°C. Energy for maintenance refers to cell survival activities such as re-synthesis of damaged cellular material, maintaining concentration gradients across the cell membrane, cell motility,… (Nystrom and Gustavsson, 1998). The concept of adding a maintenance coefficient to the maximum yield was introduced a long time ago (Pirt, 1965). It has also been postulated that the maximum yield is affected by environmental factors such as temperature, pH and osmotic pressure (Metcalf and Eddy, 2003). The effect of pH has been experimentally demonstrated, with lower than optimal pH matching with slightly lower maximum yield values (Blackburne et al., 2007a). This study indicates that the temperature effect is also valid, although a more extensive screening at different temperatures should provide more knowledge in order to derive a relationship between temperature and maximum yield. It should also be taken into account that to safeguard the membrane stability and permeability, bacteria can adjust the chemical composition and thus adapt to fluctuating temperatures (Koga, 2012). Although seasonal temperature variations can be high in moderate climates (10–25°C), the slope of this change is very low (0.07°C d^-1^) (Gilbert et al., 2015). These rather small daily changes might, thus, not even influence the maximum yield because sufficient time passes to enable adaptation toward the higher/lower temperatures. It is, thus, possible that the observed effect of temperature on the nitrifier yield would not occur in existing wastewater treatment plants, as such temperature shocks (15–28°C) would not occur within hours.

### Validation of Optimal Experimental Conditions Using Calculations and Parallel Activity Tests

**FIGURE 6:**
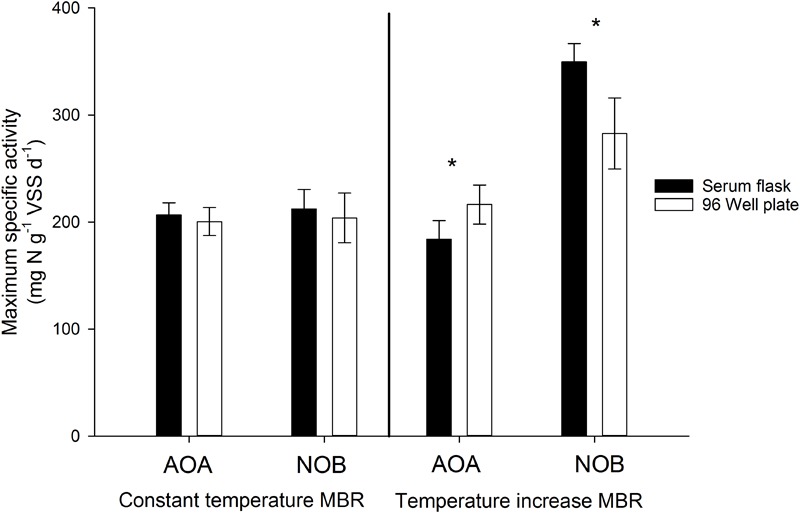
Maximum specific activity of AOA and NOB during the ex-situ activity measurements in serum flasks vs. 96 Well plate using thermophilic nitrifying biomass from the constant temperature MBR and the temperature increase MBR (Courtens et al., 2016a,b). Error bars represent the standard deviation of triplicate and sextuple incubations of the same inoculum in serum flasks and 96 Well plates respectively (technical replicates). Significant differences between serum flask and 96 Well plate values are indicated with an asterix.

**FIGURE 7:**
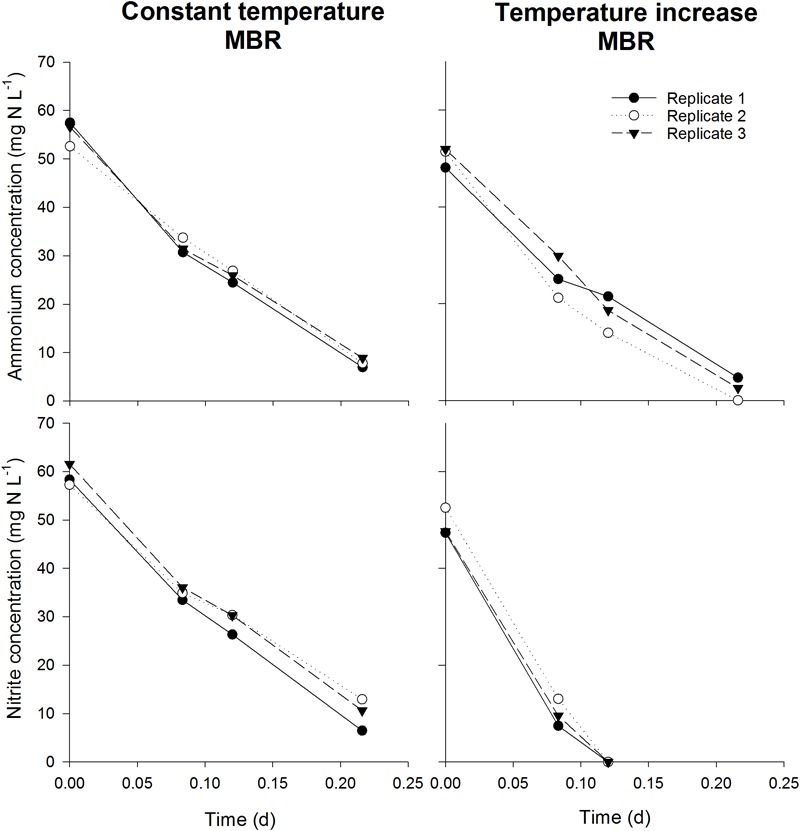
Ammonium (top) and nitrite (bottom) concentrations in the closed serum flasks (*n* = 3) during the activity measurements of the validation experiment. The linear decrease of concentration depicts the optimal conditions and non-limiting oxygen concentration. Too few measurements were available for reliable NOB rate determination in the “temperature increase MBR” incubation. However, the aim of this experiment was to determine possible oxygen limitation, which is demonstrated.

As the serum flasks were closed during incubation, it was important to assess whether sufficient oxygen was present in the flasks to ensure the complete oxidation of the substrate and concomitant incorporation of ^13^C. For this, the amount of oxygen required to oxidize the added substrate was calculated, which was compared to the calculated amount of oxygen present in the headspace and in the oxygen saturated mixed liquor at the imposed temperatures (assuming sufficiently fast transfer of oxygen between headspace and liquid). In all cases, an excess of oxygen was present in the serum flask (factor 1.58–1.79). Furthermore, again assuming a fast transfer of oxygen to the liquid, the oxygen concentration in the liquid after oxidizing all substrate was calculated using the available oxygen, the consumed oxygen and the Henry constant at the imposed temperatures. Oxygen concentrations between 2.2 and 5.1 mg O_2_ L^-1^ were obtained, which are well above reported oxygen affinity values of nitrifying organisms. Although endogenous respiration by autotrophs and heterotrophs was not taken into account, these calculations indicate that oxygen was not limiting during the incubation. To further ensure oxygen was not limiting during the experiments in closed serum flasks, a serum flask activity test was performed in parallel with an activity measurement in 96 well plates. 96 Well plates for activity measurements has been used and validated before, ensuring optimal conditions and no oxygen limitation (Courtens et al., 2016a,b). In general, maximum specific activities obtained in the serum flasks and the 96 well plate were not significantly different (*p* > 0.05), indicating that sufficient oxygen was present in the closed serum flask for complete nitrogen oxidation (Figure 6). However, for the AOA and NOB in the temperature increase MBR, maximum specific NOB activity measured in the serum flasks was higher (*p* < 0.05) than the value acquired in the 96 Well plate, which actually strengthens the notion of non-limiting oxygen conditions in the serum flasks. Further evidence that sufficient oxygen was available is depicted by linear decrease in nitrogen concentrations during the activity measurements (*R*^2^ > 93%) (Figure 7). If oxygen would become limiting, the decreasing activity would be visible in the nitrogen concentration profile and the linear decrease would falter.

### Sensitivity Analysis of the Developed Method

**FIGURE 8:**
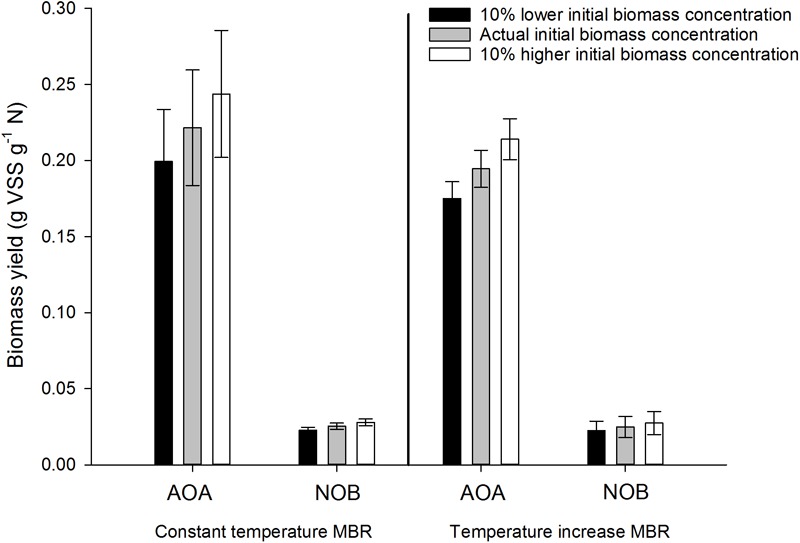
Sensitivity analysis of the obtained biomass yield for AOA and NOB in the constant temperature and temperature increase MBR in three different scenarios: the actual initial biomass concentration, a 10% higher initial biomass concentration and a 10% lower initial biomass concentration. Error bars represent the standard deviation of triplicate incubations with the same inoculum (technical replicates).

Measurements are prone to error, which might affect the result obtained in the end. For this method, the highest error can be expected in determining the initial biomass concentration. This concentration could have a major impact on the result because it is the start of all calculations and the method used to determine the biomass concentration is known to be somewhat prone to error. During reactor operation of the thermophilic inocula, triplicate measurements (technical replicates) have shown errors between 0.6 and 9.9%. The spectrophotometric method for ammonium and nitrite on the other hand is precise and less prone to error and the measurement of ^13^C is a very precise method with errors in the range of 0.0005%. Therefore, only an error on the initial biomass concentration was imbedded in the sensitivity analysis. To investigate the effect of an error in the biomass concentration, a 10% lower or higher initial concentration was assumed in the results of both thermophilic nitrifying inocula (Figure 8). No significant impact was observed, the newly developed method is thus precise and reliable for the determination of the yield of nitrifying organisms.

The incubations contained between 1 and 1.2 g VSS L^-1^ of biomass, of which about 53% carbon (C H_7_O_2_N) that was primarily in the form of ^12^C (a^13^C = 1–1.1%). When biomass decay occurs, ^12^C is released in the medium, diluting the ^13^C_medium_. This dilution impacts the calculations, namely (Equation 1), where a^13^C_medium_ is taken into account. The impact of the decay on the dilution of a^13^C_medium_ and on the Y_max_ was evaluated in two scenarios.

The first scenario considers the death-regeneration concept from the ASM models (Henze et al., 2000). In this concept, decay converts biomass to a combination of particulate matter (8%) and slowly biodegradable substrate (92%). The slowly biodegradable substrate is then hydrolyzed and becomes available for heterotrophic biomass to feed and grow upon. A part of the dead biomass ends up in new heterotrophic biomass, while the other part is oxidized to ^12^CO_2_ and ^13^CO_2_. The decay rate at 15°C was 0.15 d^-1^ (Henze et al., 2000), at 28°C 0.38 d^-1^ and at 50°C 0.4 d^-1^ (Vandekerckhove et al., 2018). A mesophilic biomass yield of 0.67 g COD g^-1^ COD (Henze et al., 2000) and a thermophilic biomass yield of 0.75 g COD g^-1^ COD (Vandekerckhove et al., 2018) was assumed. The Y_max_ in this scenario was very similar to the Y_max_ obtained as such (without taking into account the dilution of ^13^C_medium_) (Table 5). This scenario is the most probable scenario, showing that the obtained results were reliable.

**Table 5:** AOB/AOA and NOB yield of a mesophilic nitrifying inoculum at 15 and 28°C and of two thermophilic nitrifying communities at 50°C, determined as such, corrected for the dilution of 13C_medium_ by biomass decay according to the death-regeneration concept and the worst-case scenario where all dead biomass was converted to CO_2_.

	As such (g VSS g^-1^ N)	Death-regeneration (g VSS g^-1^ N)	All decay to CO_2_ (g VSS g^-1^ N)
**15°C**			
AOB	0.11 ± 0.01	0.12 ± 0.02	0.16 ± 0.02
NOB	0.051 ± 0.002	0.051 ± 0.002	0.080 ± 0.003
**28°C**			
AOB	0.06 ± 0.01	0.06 ± 0.01	0.07 ± 0.01
NOB	0.048 ± 0.001	0.053 ± 0.001	0.070 ± 0.001
**50°C–MBR_T,Constant_**			
AOA	0.22 ± 0.04	0.23 ± 0.04	0.25 ± 0.05
NOB	0.025 ± 0.002	0.026 ± 0.002	0.029 ± 0.002
**50°C– MBR_T,increase_**			
AOA	0.22 ± 0.01	0.23 ± 0.01	0.27 ± 0.02
NOB	0.028 ± 0.008	0.028 ± 0.008	0.032 ± 0.010

The second and worst-case scenario assumed that all dead biomass was converted to CO_2_ with a^13^C_biomass_ of 1–1.1%. Decay in this scenario was biomass consuming internal carbon to gain maintenance energy, without cell lysis and release of substrate for other heterotrophs. The same decay rates were assumed as in the first scenario. In this scenario, the Y_max_ was slightly different from the Y_max_ determined as such. The most pronounced effect was on the Y_max,AOA_ and Y_max,NOB_ at 15°C because that incubation (21.5 h) lasted longer than the incubation at 28°C (8 h) and 50°C (4 h). The Y_max,AOA_ at 50°C in this scenario was 1.5 times higher than the Y_max,AOB_ at 15°C instead of 2 times higher. The main conclusion, that thermophilic nitrifier yield is higher than its mesophilic counterpart, still applied in this scenario. Also, this is the worst-case scenario, which is less likely to occur than the first scenario.

### Implications for Modeling

Although cell maintenance and its effect on the maximum yield has been scientifically proven, it is not embedded in models for wastewater treatment. The maximum autotrophic yield (Y_A_) is fixed, in the ASM models at a value of 0.17 g VSS g^-1^ N, or 0.24 g COD g^-1^ N (Henze et al., 2000). Under the hypothesis that this might not suffice and that the maximum autotrophic yield value has a large impact on WWTP operation, several simulations with different autotrophic yield values were run. In order to place the results in the relevant context, some indicative process variables are provided (Table 6).

**Table 6:** Indicative process variables for the two considered cases.

Parameter	Potato wastewater	Municipal wastewater	Unit
HRT	0.55	1.54	d
SRT	21.0	16.9	d
COD/N in aerobic basin	5.9	6.2	–
Biomass specific nitrogen loading rate	0.023	0.069	g N g^-1^ VSS d^-1^
Biomass specific COD loading rate	0.140	0.424	g COD g^-1^ VSS d^-1^
Food to biomass ratio for COD	0.070	0.133	–
Biomass concentration	4258	3500	g TSS m^-3^

**FIGURE 9:**
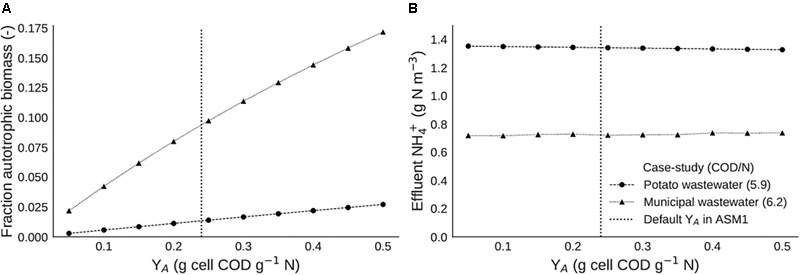
Steady state scenario analysis with different values of nitrifier yield (Y_A_), with **(A)** the autotrophic biomass fraction and **(B)** the effluent ammonium concentration.

An increase in autotrophic yield causes an increase in autotrophic biomass fraction in both cases (Figure 9A). This is in line with expectations, as a higher amount of biomass produced per amount of nitrogen would indeed lead to more nitrifying biomass. The fact that the autotrophic fraction is higher in the municipal case is explained by the higher nitrogen loading rate. In terms of effluent ammonia, the value of Y_A_ seems to have no impact in any of both cases, effluent ammonia concentrations remain constant over the whole range of Y_A_ values simulated (Figure 9B).

Overall, when the actual Y_A_ would be different from the fixed value in the classic ASM models, the simulated case studies indicate that an extension of the Y_A_ value with a maintenance factor is not essential for a correct prediction of effluent concentrations. This means that some microbiological features need not be included in modeling to obtain accurate predictions of an engineered system, avoiding too complex models.

## Author Contributions

TV, SB, SV, and NB designed the experiments. TV executed the experiments. CD designed the model and performed the model simulations. TV, SB, CD, SV, and NB analyzed and interpreted the data, wrote and edited the manuscript.

## Conflict of Interest Statement

The authors declare that the research was conducted in the absence of any commercial or financial relationships that could be construed as a potential conflict of interest.
